# Multi-organ pathology in a small porcine model of cytokine storm syndrome characteristics

**DOI:** 10.3389/fimmu.2025.1618665

**Published:** 2025-07-23

**Authors:** Jiaqian Jin, Linxiao Fan, Richai Chen, Xuanhao Lin, Sainan Zhang, Mengfei Zhu

**Affiliations:** ^1^ Zhejiang Chinese Medical University, Hangzhou, China; ^2^ Key Laboratory of Artificial Organs and Computational Medicine in Zhejiang Province, Shulan (Hangzhou) Hospital Affiliated to Shulan International Medical College, Zhejiang Shuren University, Hangzhou, China

**Keywords:** cytokine storm syndrome (CSS), D-galactosamine (D-GalN), liver failure, interleukin-6 (IL-6), histopathology

## Abstract

**Introduction:**

Cytokine Storm Syndrome (CSS) is a severe immune dysregulation characterized by excessive cytokine release and multi-organ injury. Existing murine models inadequately replicate human CSS. This study aimed to establish a D-galactosamine (D-GalN)-induced miniature pig model to investigate multi-organ pathological changes and inflammatory dynamics.

**Methods:**

Twenty-four male minipigs were divided into control and D-GalN groups (n=12 each). The experimental group received 1.5 g/kg D-GalN intravenously. At 0 h, 12 h, 24 h, and 36 h post-induction, tissues from the liver, lungs, intestines, heart, spleen, and kidneys were collected for hematoxylin-eosin (H&E) staining and IL-6 immunohistochemistry.

**Results:**

Histopathology revealed progressive, time-dependent multi-organ injury. The liver, lungs, and intestines showed the earliest and most severe damage, including hepatocellular necrosis, alveolar congestion, and intestinal epithelial destruction. IL-6 expression increased over time, first peaking in the liver and later spreading to the lungs, intestines, and other organs. At 36 h, IL-6 was diffusely expressed in hepatic and pulmonary tissues, indicating an escalating systemic inflammatory response.

**Discussion:**

This minipig CSS model replicates human-like disease progression and identifies the liver as a likely initiator of systemic inflammation. The observed “liver initiation–lung and intestine diffusion” pattern provides new insights into CSS pathogenesis. The temporal expression of IL-6 suggests a critical therapeutic window prior to 24 h post-onset for anti-inflammatory interventions, including artificial liver support and IL-6 blockade.

## 1 Background

CSS is an immune dysregulation syndrome triggered by factors such as infections, malignancies, or autoimmune diseases (1). It is characterized by a marked elevation of multiple cytokines in plasma, accompanied by fever, multisystem or multiorgan inflammation, and functional impairment. Cytokines regulate inflammatory responses, immune cell activation, and tissue repair through a complex network. Dysregulation of this network can lead to CSS. Among these cytokines, IFN-γ, IL-1, IL-6, TNF, and IL-18 are recognized as the major pathogenic mediators (2, 3). Currently, there is no universally accepted definition of CSS. To enhance conceptual clarity, this article summarizes its common etiologies, key mediators, and clinical consequences (Refer to **Table 1**).

**Table 1:** Summary table of cytokine storm syndrome (CSS).

Category	Details
Common Triggers	• Infections: Bacteria, viruses (e.g.SARS-CoV-2, influenza) • Autoimmune diseases: SLE, rheumatoid arthritis • Tumor-related: CAR-T therapy • Trauma: Severe tissue injury, burns
Key Cytokines	• IL-1β, IL-6, TNF-α, IFN-γ, IL-18
Other Mediators	• C-reactive protein (CRP) • Coagulation factors • Platelet-activating factor • Complement proteins
Pathophysiological Mechanisms	• Dysregulated immune activation → massive cytokine release (1) • Increased vascular permeability → Organised oedema (4) • Microthrombi formation → circulatory disturbance (5)
Clinical Consequences	• Fever, hypotension • Organ dysfunction (lung, liver, kidney) • Multiple organ failure (MOF) • High mortality

This table is adapted from Fajgenbaum DC, June CH. Cytokine Storm. New England Journal of Medicine (2).

Although mice are widely used in the construction of cytokine storm models due to their well-defined genetic background, low cost, and short reproductive cycle, a study by Seok et al. (6)demonstrated significant limitations in their ability to mimic human CSS. These include poor correlation in gene expression, differences in signaling pathway regulation, and insufficient complexity of immune responses. In contrast, Zurek-Leffers et al. (7)found that the porcine immune system closely resembles that of humans in both structure and function, particularly regarding innate and adaptive immune effectors, making it more comparable. In pigs, the dynamic changes of inflammatory cytokines—such as IL-6—during CSS are consistent with those observed in humans, and elevated IL-6 levels are strongly associated with disease severity and prognosis. Moreover, pigs can exhibit human-like vascular endothelial dysfunction during inflammatory states, including increased vascular permeability and fluid leakage. Therefore, this study employs miniature pigs as the animal model for cytokine storm in order to more accurately replicate the pathogenesis of human CSS.

**Figure 1:**
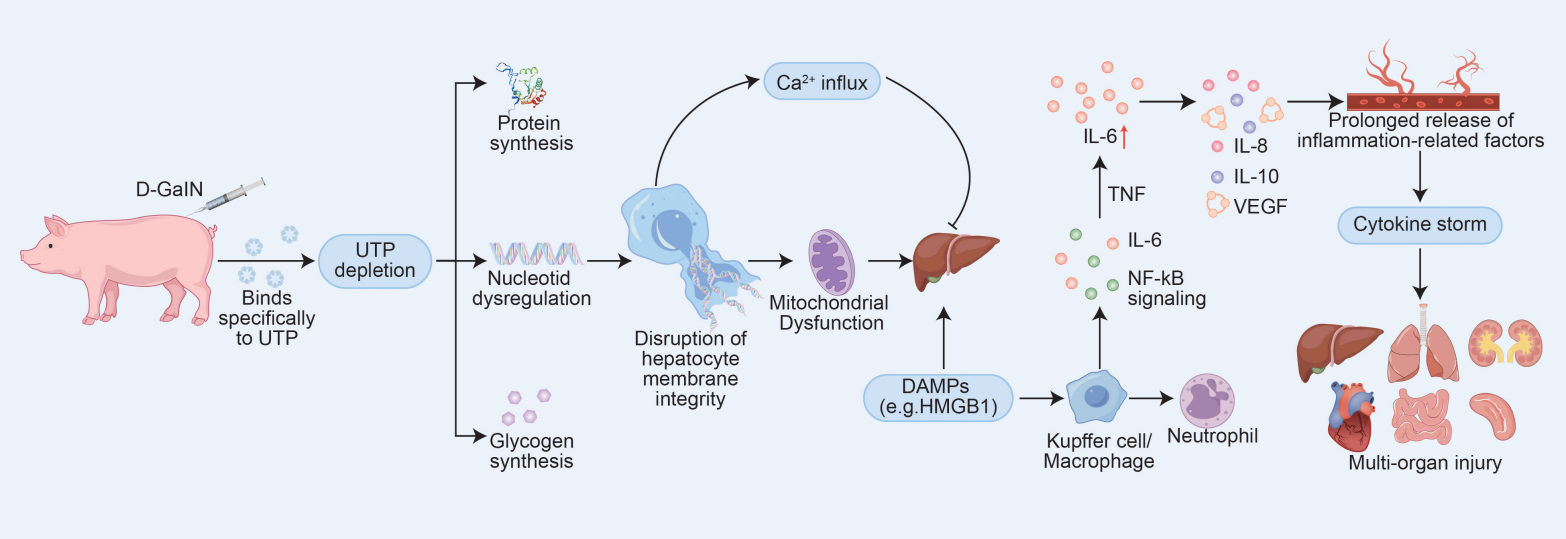
D-GalN-Induced Hepatotoxic Pathways and Their Role in Initiating CSS. The mechanism diagram was constructed based on data and models described in references (9, 10, 13) and (14).Abbreviations: UTP, uridine triphosphate; IL-6, interleukin-6; IL-8, interleukin-8; IL-10, interleukin-10; VEGF, vascular endothelial growth factor.

Based on previous research, we selected D-galactosamine (D-GalN) as the modeling agent. D-GalN binds with uridine triphosphate (UTP) to form UDP-GalN and inhibits UDPG pyrophosphorylase through phosphorylation pathways, leading to significant depletion of UTP and UDPG. This inhibition suppresses the synthesis of nucleic acids, proteins, and glycogen in hepatocytes, disrupts cellular membrane systems, induces intracellular Ca²^+^ influx, and ultimately causes hepatocellular necrosis (8). This mechanism enables the stable and reproducible establishment of liver failure animal models (9), with a relatively predictable progression once modeling is successful. More importantly, liver failure induced by D-GalN can compromise the intestinal barrier, allowing translocation of gut microbiota and toxins into the bloodstream, which may lead to spontaneous peritonitis, bacteremia, and systemic inflammatory responses (10). The acute phase of infection is often accompanied by injury to multiple organs such as the kidneys and lungs, as well as features characteristic of CSS (11, 12). Therefore, we consider D-GalN an ideal agent for constructing an animal model of CSS. In this study, the occurrence of CSS was inferred indirectly through time-dependent histopathological changes and patterns of multiorgan damage. Coupled with extensive validation of this model in the literature, our findings support D-GalN-induced liver failure as an effective animal model for studying CSS. To better illustrate its metabolic dysregulation and inflammatory mechanisms, **Figure 1** has been created.

In the treatment of CSS, identifying the etiology of the disease and implementing targeted therapies (e.g., anti-infective, anti-tumor) is the first priority, while regulating the uncontrolled immune response is also critical. Currently, commonly used interventions include hormones, cytotoxic drugs (e.g., cyclophosphamide), interleukin inhibitors (e.g., tolizumab, an IL-6 inhibitor) (15), and artificial liver purification therapy (1). However, the optimal timing of intervention for these interventions is unclear. Studies have shown that anti-IL-6 therapy may be more effective in the early stages of inflammation, but should not be used too early to avoid interfering with antiviral immunity (16). Although several guidelines emphasize the potential value of hormones and artificial liver in rapidly controlling inflammation, clearing cytokines, and reducing morbidity and mortality, the therapeutic window still lacks uniformity.

Therefore, this study aims to investigate the timing of multiorgan injury in D-GalN-induced CSS in model minipigs from the perspective of multiorgan histopathology, in order to provide a rationale and supporting evidence for determining the optimal timing of internal medicine and artificial liver blood purification interventions in patients with CSS.

## 2 Experimental objective

This study aims to investigate the pathological changes in multiple organs during the development of CSS induced by D-GalN, as observed through histopathological analysis. By systematically analyzing the pathological features of each organ, the study seeks to identify the key time points of these changes, thereby providing important evidence for the early diagnosis and intervention of CSS.

## 3 Experimental methods

### 3.1 Experimental materials

#### 3.1.1 Experimental animals

Twenty-four male castrated Parma pigs weighing 15-25kg were kept in the Laboratory Animal Center of Zhejiang Institute of Traditional Chinese Medicine, all animals were kept in single cages and fed with special feed for suckling pigs. The animals were routinely kept for 3 days before the experiment to adapt to the environment. The animals were forbidden to eat and drink 12h before jugular vein cannulation.

#### 3.1.2 Main drugs and reagents

Povidone-iodine, tiletamine hydrochloride for injection, zolazepam hydrochloride (Shutex 50), D-aminogalactosamine (D-GALN), heparin, saline, 5% dextrose, formalin, hematoxylin-eosin staining, interleukin-6 antibody, interleukin-10 antibody, micropore filter membrane.

### 3.2 Research methods

#### 3.2.1 Configuration of D-aminogalactose hydrochloride solution

Weigh 50g of D-GalN hydrochloride powder, dissolve it in 100ml of 5% dextrose injection, and prepare a solution of 0.5g/ml, use 0.22um microporous filter membrane to filter and decontaminate the configured solution and refrigerate it, and use it within 2 hours.

#### 3.2.2 Jugular vein cannulation

Select the Parma pigs to be intubated, and the animals were fasted and dehydrated 12h before the start of the experiment. Anesthesia was induced by intramuscular injection of Sutex 50 (125mg/20kg). After the animals were anesthetized, they were fixed on the operating table in the left lateral position. The skin of the right neck was prepared, sterilized with iodophor and a surgical cavity towel was spread. The skin was incised along the right temporomandibular joint to 1 cm below the line of the acromion with a scalpel, and the skin was incised from the temporomandibular joint toward the acromion. The connective tissue was separated in the middle curvature, the common carotid artery was exposed, and the trunk of the vessel about 3 cm long was freed. The distal end was ligated with a surgical suture, and another suture was taken through the proximal end but not ligated. The vein was flattened with ophthalmic forceps, a descending wedge-shaped incision was cut, and a double-lumen anti-infective Arrow tube was inserted through the vascular incision to a depth of 0.5–1 cm above the exposed skin.The catheter was injected with 1:125 sodium heparin to seal the tube and to prevent coagulation in the lumen. The proximal end was ligated with thread wrapped around the epithelium and the Arrow tube, the wound was flushed with iodine povidone, and the muscular layer was closed with continuous sutures and a skin stapler. Butterfly clips were used to secure the double lumen catheter, both side holes were sutured, heparin cap was used to seal the orifice, the incision was covered with sterile gauze, and the neck was wrapped with an elastic bandage. The whole operation was completed within half an hour, and when the operation was finished and the animals were awakened, they were sent to the rearing room to be reared in separate cages.

#### 3.2.3 Animal model induction

Reanesthetize the minipigs with Sutent 50 (125mg/20kg), weigh them, and draw the configured D-GaLN solution at a dosage of 1.5g/kg, and slowly push the model through the double-lumen Arrow tube silently.

#### 3.2.4 Experimental grouping

**Figure 2:**
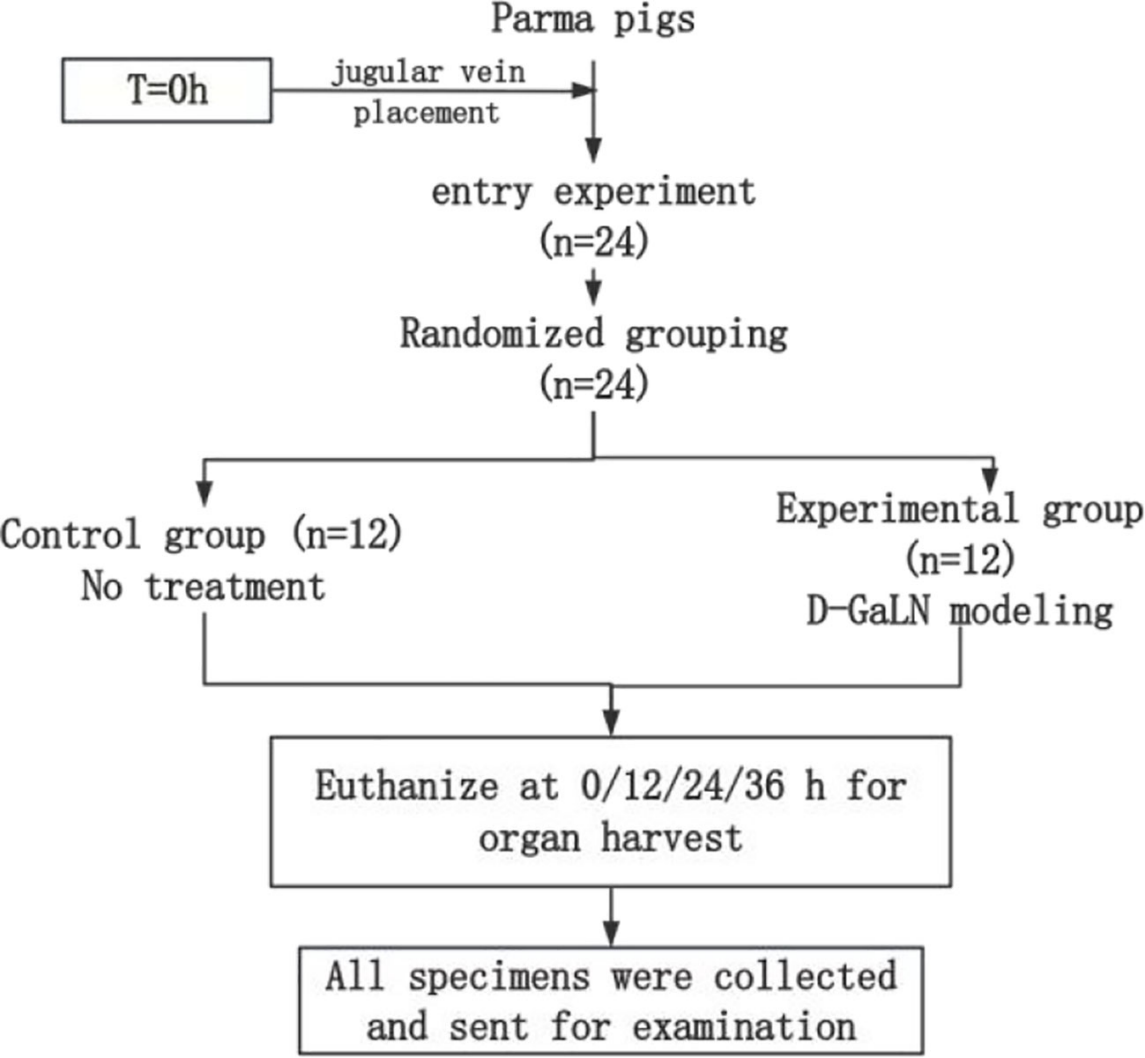
Experimental groups and procedure.

The entire experimental process is shown in **Figure 2**. After jugular vein cannulation, 24 Parmesan pigs were divided into the following two groups by the random draw method and started to receive different interventions.

(1) Control group (n=12) Without any treatment, 3 pigs were executed by air injection to take organs (including heart, liver, spleen, lungs, kidneys, and intestines) at 0h, 12h, 24h, and 36h after modeling, respectively, and the pathological specimens taken were fixed in formalin solution;
(2) Experimental group (n=12) D-GaLN modeling was performed at 0 h. The specific modeling method was described in 1.2.3. Execution, pathology and fixation were performed at the same time as that of the control group, with the same process and requirements as that of the control group.

Specimens from each organ were sent to the Department of Pathology for Hematoxylin and Eosin (HE) staining and IL-6 immunohistochemical staining.

### 3.3 Analysis of pathological results

All the pathological HE and IL-6 sections in this study were read by two physicians with the title of deputy chief physician or above in the Department of Pathology and unified diagnostic opinions.

## 4 Results

### 4.1 Histopathological changes in HE staining of multi-organ tissues of minipigs with CSS

**Figure 3:**
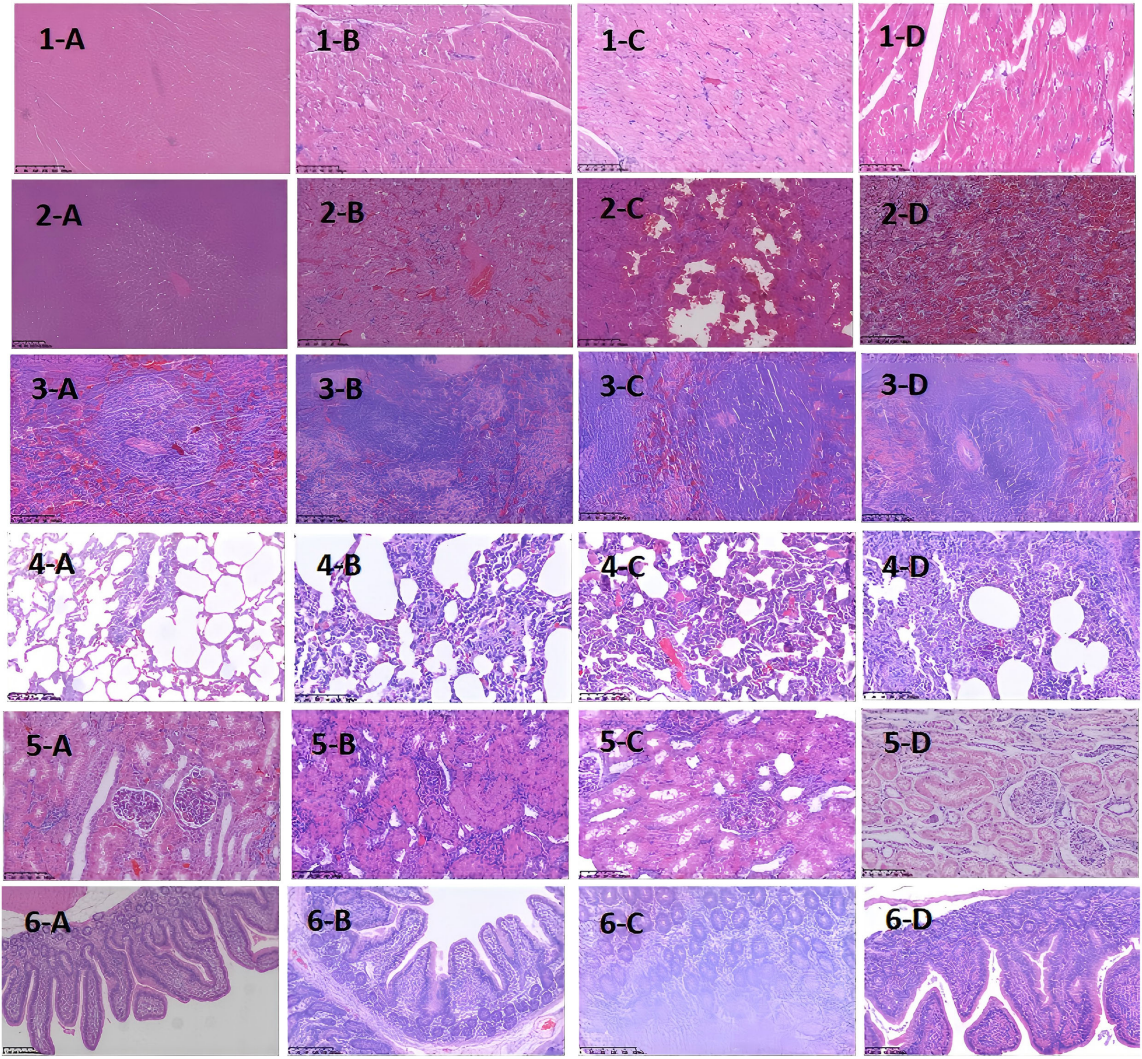
Histopathological Images of Major Organs at Different Time Points After D-GalN Induction (H&E Staining). Multi-organ pathology. Staining with hematoxylin and eosin (H&E) shows different organ pathology maps at different sampling time points after modeling with D-GaLN. **(A-D)** represent 0 h, 12 h after modeling, 24 h after modeling, and 36 h after modeling, respectively.1–6 represent the heart, liver, spleen, lungs, kidneys, and intestines, respectively.D-GaLN-induced cytokine storms resulted in multi-organ damage.

As the modeling time progressed, pathological damage in various organs gradually worsened, showing a clear temporal pattern. At 12 hours after modeling, significant pathological changes first appeared in the liver and intestines. By 24 hours, damage to the liver, lungs, and intestines had further intensified. At 36 hours, pathological injury peaked in multiple organs (liver, heart, lungs, and intestines), characterized by extensive cell necrosis, inflammatory cell infiltration, and disruption of tissue structure. The spleen and kidneys showed relatively milder pathological changes but still exhibited varying degrees of congestion and swelling. To more clearly and intuitively present the histological changes of each organ at different time points, we summarized the main pathological findings in **Table 2**, while **Figure 3** displays the representative histopathological features of each organ.

**Table 2:** Histopathological changes in major organs at different time points after D-GalN induction (H&E Staining).

Organ	12h	24h	36h
Liver	Disrupted hepatocyte arrangement, sinusoidal endothelial cell injury	Hepatocyte congestion and necrosis, aggravated central vein and sinusoidal congestion	Extensive hepatocyte necrosis, hydropic degeneration, vacuolar steatosis, moderate to severe lobular congestion
Intestine	Infiltration of eosinophils, shortened villi, shallow crypts	Massive lymphocyte infiltration in mucosa, mucosal erosion	Dense eosinophil and lymphocyte infiltration, vascular endothelial shedding
Lung	Intact bronchial mucosa, no significant alveolar abnormalities	Alveolar wall congestion, edema fluid accumulation, capillary thrombosis	Massive neutrophil infiltration in bronchi and alveoli, lobar pneumonia formation
Heart	Mild edema around serosal vessels, slight endocardial edema	Subendocardial edema more pronounced than at 12h	Extensive inflammatory cell infiltration in serosa, marked exudation, persistent endocardial edema
Spleen	Normal structure	Overall normal; mild inflammatory changes in red pulp and vascular areas	nflammatory cell infiltration in red pulp and vascular intima, moderate inflammation in white pulp; structure largely preserved
Kidney	Moderate to severe swelling of proximal tubules	Moderate to severe swelling of proximal tubules	Mild swelling of proximal tubules; glomerular structure remains intact

### 4.2 Pathological changes in immunohistochemical staining of IL-6 in multiple organ tissues of minipigs with CSS

**Figure 4:**
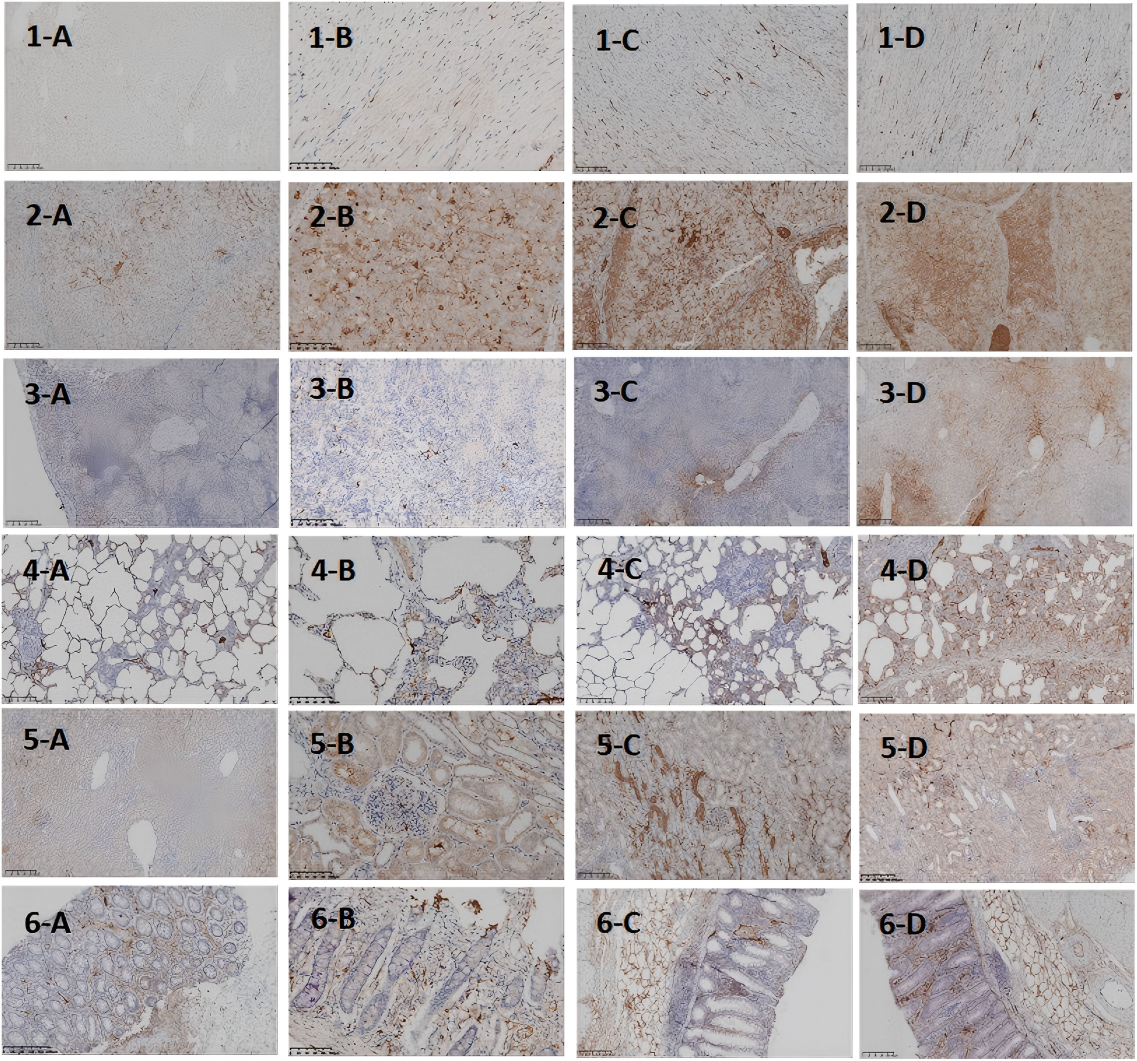
Histopathological Images of Major Organs at Different Time Points After D-GalN Induction (IL-6 Staining). IL-6 expression at different time points and in different organs in the DaLN-induced cytokine storm model. **(A-D)** represent 0h, 12h after modeling, 24h after modeling, and 36h after modeling, respectively.1–6 represent heart, liver, spleen, lungs, kidneys, and intestine, respectively.

As the modeling time progressed, the distribution and expression of IL-6 in different organs underwent significant changes. Overall, IL-6 expression gradually expanded from initially localized and sparse areas to broader regions, with a marked increase in expression levels. Notably, IL-6 expression was significantly elevated in the liver and lungs, indicating an intensifying inflammatory response over time. To more intuitively illustrate the changes in IL-6 expression across different organs and time points, the relevant results are summarized in **Table 3**, with representative pathological images shown in **Figure 4**.

**Table 3:** Temporal changes in IL-6 expression in major organs after D-GalN induction (Immunohistochemistry).

Organ	12h	24h	36h
Liver	Some IL-6 deposition in cells; no expression in membrane or cytoplasm	Widespread expression in hepatic lobules (about 60%–70% area)	Diffuse nuclear and cytoplasmic expression, >80% area
Intestine	Slightly increased expression (about 5% area)	Similar to 12h	Marked increase in mucosal stroma expression (about 30% area)
Lung	Increased interstitial expression (about 30%–40%); no alveolar expression	Similar to 12h	High diffuse expression in bronchial lumen, alveolar epithelium, interstitium, and endothelium (about 50%–60%)
Heart	No myocardial expression; minor IL-6 perfusion in vessels (about 10% area)	Same as 12h	Same as 12h
Spleen	IL-6 distributed around splenic sinusoids (about 3%–5% area)	High expression in red pulp and vascular intima	Moderate expression in white pulp; strong expression in red pulp and vascular areas
Kidney	Minor periglomerular IL-6 expression in inflammatory cells	Widespread expression in distal tubules (about 15% area)	Increased expression in proximal/distal tubules, glomerular endothelium, and renal arterial intima (about 20% area)

## 5 Discussion

CSS is a severe immune response dysregulation phenomenon, which is centrally characterized by a dramatic increase in plasma cytokine levels, triggering a systemic inflammatory response and leading to multi-organ or multi-system damage, which, if not treated promptly and effectively, may further progress to multi-organ failure. In the treatment of cytokine storm syndrome, the key to prognosis lies in the precise regulation and control of excessive immune responses *in vivo*. Therefore, the successful construction of a D-GalN-induced minipig model is extremely important research value for the in-depth study of immune intervention in CSS and the optimal timing of blood purification therapy.

Although the traditional D-GalN mouse model can simulate fulminant hepatic injury (progressing to liver failure within 6–8 hours), its extremely short survival time (with 90% of mice dying within 12 hours) severely limits the study of secondary damage mechanisms in extrahepatic organs (17). In contrast, the minipig model used in this study presents three major advantages: first, its hepatic drug-metabolizing enzyme profile, physiological parameters, and body size are more comparable to those of humans (18); second, the liver injury progresses gradually with disease onset, better reflecting the clinical trajectory of liver function deterioration in CSS patients (19); and most importantly, this controllable time window provides the possibility for evaluating interventions such as artificial liver support.

Through organ sampling and HE staining analysis of minipigs after modeling, we observed that the systemic inflammatory response triggered by CSS caused widespread damage to multiple organs. Six key target organs — the heart, liver, spleen, lungs, kidneys, and intestines — all exhibited varying degrees of pathological inflammatory injury, with the liver, lungs, and intestines being the most severely affected.At 24 hours after modeling, pathological analysis revealed congestion in the hepatic lobules, along with small localized foci of necrosis. The liver performs non-immune functions such as metabolism, nutrient storage, and detoxification, and is also a key component of the immune system. It is responsible for the synthesis of acute-phase proteins, complement components, cytokines, and chemokines, and contains a diverse population of resident immune cells (20). The proper functioning of these inflammatory mechanisms is crucial for maintaining liver homeostasis, defending against pathogens, inhibiting tumor growth, and repairing tissue damage. However, when the immune response becomes excessive, this homeostasis is disrupted, leading to altered hepatic hemodynamics, increased capillary permeability, enhanced leukocyte infiltration into tissues, and excessive production of inflammatory mediators (21).At the same time point (24 hours after modeling), the lungs exhibited pathological changes such as alveolar septal congestion, alveolar edema, and capillary thrombosis. As CSS progressed, the pathology showed a gradual increase in neutrophil infiltration within the bronchi. By 36 hours after modeling, the alveolar spaces were filled with large numbers of neutrophils, ultimately leading to lobar pneumonia. Another severely affected target organ, the colon, showed destruction of villous structures and marked loss of crypts on HE staining. Additionally, massive infiltration of neutrophils and plasma cells was observed in the intestinal mucosa. These pathological changes led to dysfunction of the intestinal mucosal barrier, allowing large numbers of antigenic molecules and microorganisms to translocate into the body via the paracellular pathway, further exacerbating the inflammatory response.

To further clarify the relationship between target organ damage and the extent of IL-6 infiltration in the progression of CSS, we performed IL-6 immunohistochemical staining of pathological tissues from the above organs. The results showed that at the four time points of organ sampling, the extent of hepatic IL-6 infiltration gradually expanded with the prolongation of modeling time, and by 36 h, IL-6 was diffusely expressed in the nucleus and cytoplasm in >80% of the area.This significant concentration of inflammatory signals suggests that the liver may be the first to be activated in the immune storm of the CSS, assuming the role of the “starting point” for driving the systemic inflammatory cascade. This suggestion is also supported by the clinical case shared by Bian XW et al. (22)in the autopsy of a critically ill patient with COVID-19, in which the characteristic necrosis of the central hepatic lobules with neutrophilic infiltration was highly consistent with the 36h hepatic pathology induced in the minipig model of the present study by D-GaLN.The timing of injury—such as hepatocyte disorganization and sinusoidal endothelial damage observed at 12 hours post-modeling—supports the conclusion by Wang H et al. (23) that hepatocytes are not mere “victims” of inflammation, but “initiators” actively amplifying the systemic inflammatory storm. To better understand the mechanistic aspects of this initiator role, we reviewed studies on the liver’s involvement in the CSS inflammatory cascade. The liver may amplify the inflammatory response in CSS through multiple synergistic mechanisms. First, hepatic macrophages (Kupffer cells) are highly sensitive to damage-associated molecular patterns (DAMPs), making them a key source of early IL-6 release (13, 14). Second, the liver’s high-flux blood flow facilitates the rapid systemic dissemination of inflammatory mediators, forming an “initiation-diffusion” pattern. Intrahepatic natural killer (NK) cells also secrete large amounts of IFN-γ and activate the STAT1 signaling pathway, further enhancing macrophage-driven inflammation (24). In addition, D-GalN, as a specific hepatotoxicity inducer, causes hepatocyte necrosis and triggers a localized inflammatory response. As inflammation expands, large quantities of cytokines and chemokines enter the circulation, triggering CSS and eventually leading to multi-organ dysfunction (25).We observed varying degrees of IL-6 infiltration and tissue damage in both lung tissue and intestines 24 hours after D-GalN-induced modeling.Damage to the intestinal epithelial structure was accompanied by diffuse IL-6 expression, suggesting that the feedback activation mechanism of the hepatic-intestinal axis may play a key role in CSS. Meanwhile, IL-6 expression was significantly enhanced in lung tissues. It has been shown that IL-6 can induce immune cells to accumulate in the lungs and release free radicals and proteases, which can damage the alveolar epithelium and capillary endothelium; at the same time, IL-6 can cooperate with inflammatory vesicles to promote the death of alveolar cells, which can exacerbate the destruction of lung tissue (26).Therefore, we hypothesize that the large amount of IL-6 released during hepatic inflammation may act through the bloodstream to reach the intestines and lungs, where it triggers and amplifies local inflammatory responses—one of the key mechanisms underlying early multi-organ injury in CSS. Although the study by Joseph C and Mark D (27)concluded that the spleen serves as a key source of inflammatory cytokine release in mouse LPS or IRI models—particularly through macrophages in the white pulp, which express markedly higher levels of TNF-αcompared to other organs—we observed in the D-GalN-induced minipig CSS model that the inflammatory response in the splenic white pulp was relatively weak at 0 h and remained lower at 24 h compared to 36 h. By contrast, high levels of inflammatory cell infiltration were observed in the red pulp and marginal zones, with only moderate infiltration in the white pulp. These findings may indicate a dynamic regional shift in inflammatory activation, with the liver acting as an early-stage initiator and the splenic red pulp serving as a late-stage amplifier in the progression of CSS.In addition, it is worth noting that minipigs, as a large animal model, have an immune system that is closer to that of humans, and different species and modeling methods may explain this difference.As for the kidney, and consistent with the findings of Jason et al. (28), multiple mechanisms—including the release of inflammatory factors (e.g., IL-6, TNF-α), leukocyte activation, increased apoptosis, and microcirculatory disturbances—may act synergistically to drive the onset of renal failure. However, studies in animal models have demonstrated that pathological alterations in renal tissue do not necessarily correlate with measurable functional impairment.

In summary, the present study successfully simulated the pathological process of CSS using a D-GalN-induced minipig model, revealing the temporal progression of multi-organ damage and its underlying mechanisms. The experimental results demonstrated a time-dependent increase in organ injury, with peak IL-6 expression in tissues observed at 24 hours after modeling—closely corresponding to critical pathological time points. These findings provide a valuable reference for determining the optimal timing of clinical intervention: (1) Early intervention (0–12 hours): during this window, the cytokine storm is just beginning and has not yet caused significant organ damage. Timely suppression of cytokine overproduction can effectively limit systemic inflammation and prevent multi-organ failure. (2) Critical time point (24 hours): initiating interventions before this stage—such as administration of the immunomodulatory drug tocilizumab or blood purification therapy—may significantly reduce organ damage and improve clinical outcomes.

Furthermore, the “liver initiation–lung and intestinal diffusion” cascade pattern identified as central to the CSS inflammatory response suggests that clinical interventions should be focused on targeting liver-derived cytokine release. The artificial liver blood purification system is highly effective in removing elevated circulating levels of IL-6, TNF, and other inflammatory mediators. This makes it well-suited to counteract the liver-derived inflammatory amplification mechanism demonstrated in this model.Moreover, the diffuse expression of IL-6 within 24 hours post-modeling underscores the importance of initiating anti-IL-6 therapy prior to this time point. In addition, the systemic immune activation observed in CSS provides a rationale for the potential application of cytotoxic agents (e.g., cyclophosphamide). These drugs may help mitigate early organ damage by suppressing overactivated immune cells and warrant further validation in large animal models with extended survival durations.In conclusion, this study not only enhances our understanding of the pathogenesis of CSS, but also provides experimental evidence and theoretical support for the selection and timing of early clinical intervention strategies.

## Data Availability

The raw data supporting the conclusions of this article will be made available by the authors, without undue reservation.
